# Population-wide incidence estimates for soft tissue knee injuries presenting to healthcare in southern Sweden: data from the Skåne Healthcare Register

**DOI:** 10.1186/ar4678

**Published:** 2014-07-31

**Authors:** George Peat, Charlotte Bergknut, Richard Frobell, Anna Jöud, Martin Englund

**Affiliations:** Keele University, Staffordshire, UK; Department of Orthopedics, Clinical Sciences Lund, Lund University, Lund, Sweden; Epidemiology and Register Centre South, Skåne University Hospital, Lund, Sweden; Clinical Epidemiology Research & Training Unit, Boston University School of Medicine, Boston University School of Medicine, Boston, MA USA

## Abstract

**Introduction:**

Soft tissue knee injury is a well-established and potent risk factor for development of knee osteoarthritis. However, there is a paucity of epidemiological data from the general population. Our aim was to estimate the annual person-level incidence for a wide spectrum of clinically diagnosed soft tissue knee injuries, and their distribution by age, sex, and season.

**Methods:**

In Sweden, in- and outpatient health care is registered using each individuals’ unique personal identifier including International Classification of Diseases (ICD) 10 diagnostic code(s) as determined by physicians’ clinical examination. For the calendar years 2004–2012, we studied the population in southern Sweden, Skåne region (approx. 1.3 million). We identified residents who had at least one visit to a physician with clinically diagnosed knee ligament, meniscal, or other soft-tissue injury (S80.0, S83 and all subdiagnoses). We then calculated the mean annual incidence over the 9-year period. As a secondary objective, we investigated potential seasonal variation.

**Results:**

The annual incidence for males and females was 766 (95% CI: 742, 789) and 676 (649, 702) per 100,000 persons/year respectively. For males and females, the peak rate occurred in 15 to 19 year-olds (1698 per 100,000 men and 1464 per 100,000 women, respectively). In women, rates were lowest in the 25 to 34 year-old age range before rising again between the ages of 35 and 49 years. We found substantial seasonal variation, greatest in men, with peaks in March-May and August-October.

**Conclusions:**

The incidence of clinically diagnosed soft-tissue knee injury peaks in adolescence and emerging adulthood. However, a range of knee injuries continue to occur across the adult lifespan including at ages when osteoarthritis is typically diagnosed and managed. The potential cumulative effect on osteoarthritis progression of these injuries may warrant further investigation.

**Electronic supplementary material:**

The online version of this article (doi:10.1186/ar4678) contains supplementary material, which is available to authorized users.

## Introduction

Knee injury is a potent and potentially modifiable risk factor for knee osteoarthritis, conferring an estimated three- to six-fold increased risk of osteoarthritis later in life [1, 2]. Data are consistent with a causal interpretation in those circumstances where significant injury has clearly preceded the development of osteoarthritis. Indeed, anterior cruciate ligament transection or destabilisation of the medial meniscus are used in animal models of surgically-induced osteoarthritis [3]. The strength of association, plausible causal interpretation, and potential to be modified mean that injury prevention and rehabilitation are now key priorities within public health agendas for osteoarthritis prevention [4, 5].

There remains much about the relationship between injury and osteoarthritis that requires further investigation, including the effects on osteoarthritis incidence and progression of incurring different types of injury at different ages [6–8]. A fundamental requirement is knowledge of the rate of occurrence of knee injuries across the entire lifespan. As few as 20% of soft tissue knee injuries are presented to and managed in emergency departments and hospitals [9, 10] and 50% of all knee injuries are associated with non-sporting activities [11, 12], particularly those sustained in middle age onwards [13].

Yet most studies of the incidence of soft-tissue knee injury have tended to focus their attention either on injuries captured within a single healthcare setting (for example, emergency department [13, 14] or hospital admissions [15–17]) or in high risk subpopulations (for example, children/adolescents [17, 18], sports participants [19–21], physically active compulsory military conscripts [15, 22], active duty armed service personnel [23]) or to a particular type of injury (for example, cruciate ligament injury [17, 24, 25]. The need for population-wide estimates across multiple injury types has been identified as a gap by recent reviews [26, 27]. The incidence rates for lower limb fractures have been the subject of several previous database studies (for example, [28]). We, therefore, sought to provide occurrence estimates for a range of soft-tissue knee injuries presenting to primary and secondary healthcare for persons of all ages in a general population.

## Methods

### Data source

Our study was based in Skåne, the southernmost region in Sweden, which has a population close to 1.3 million residents (2012), roughly one-eighth of the Swedish population. In Sweden all health care consultations are recorded in region-specific databases. The Skåne Healthcare Register (SHR) holds details for primary and secondary care (including all in- and out-patient health care) linked by individuals’ unique personal identification number. The register entries include information on health care provider, date of visit, and physicians’ diagnostic codes according to the Swedish version of the International Classification of Diseases (ICD) 10 system. In Sweden all individuals are registered to a general practice. Patients do not need to attend primary care before seeing a specialist although this is the most common process. Each consultation generates data entries (for example, diagnostic code(s)) that are transferred to the SHR and which constitute the basis for reimbursement to the health care providers. Similar regulations apply to both public and private health care providers and both are easily accessed. Studies within SHR of other musculoskeletal disorders have suggested high validity of diagnostic coding [29, 30]. Approximately 90% of public primary care consultations and 99% of secondary care consultations have diagnostic codes recorded in the SHR. However, diagnostic codes recorded by private outpatient and private primary care providers are not yet automatically transferred to the SHR. Diagnostic codes recorded by professionals other than doctors are not complete and not included in this analysis. Private care provider contacts account for approximately 30% of all health care and, therefore, the denominator for the Sweden database was reduced by 30%. The derivation and empirical justification of these adjustments have been presented in earlier papers [29–31]. In brief, the adjustments were based on the proportion of consultations made in private care and, for patients consulting privately, the likelihood of repeat consultation for the knee injury also to a public health care provider was considered to be low.

The study was conducted according to the Declaration of Helsinki and approved by the Ethical Review Board of Lund University, Sweden. According to the Ethical Review Board decision and in line with Swedish law, all individuals eligible for inclusion in the study were informed in the regional news press and offered an opt-out opportunity. After the linkage, all data were analysed anonymously.

### Definition of soft-tissue knee injury

We searched all records in the SHR between 2004 and 2012 for soft-tissue knee injury contacts coded using selected ICD-10 codes. Soft tissue knee injury was defined as an in-person clinic visit in primary or secondary care to a physician with one of the following recorded diagnoses: contusion of knee (ICD-10 code: S80.0), dislocation of patella (S83.0), dislocation of knee (S83.1), tear of meniscus, current injury (S83.2), tear of articular cartilage of knee, current injury (S83.3), sprain/strain involving (fibular/tibial) collateral ligaments (S83.4), sprain/strain involving (anterior/posterior) cruciate ligaments (S83.5), sprain/strain involving other/unspecified parts of knee (S83.6), sprain/strain of knee (S83.6P) and injury to multiple structures of knee (S83.7). The Swedish Population Register is the civil registration of vital events (for example, deaths, change of address). We cross linked SHR data to determine whether the knee-injured person was a resident or not of the Skåne region at the time of knee injury, thus knee-injured non-residents were not included in the study.

### Analysis

We calculated the annual cumulative incidence of residents injured for any soft tissue knee injury and separately for each injury code. This was defined as the proportion of the registered population consulting at least once with the relevant injury code recorded during the calendar year, expressed per 100,000 registered population at the start of each calendar year. In the event of persons having multiple injury consultations within the same calendar year the first consultation was used for the overall estimate and in the analysis of seasonal variation but the first consultation for each different injury code was used for injury-specific rates. For example, a person having a consultation coded for cruciate ligament sprain (S83.5) in March followed by one in April coded as meniscal tear (S83.2) would be included once in the overall annual incidence estimate (type of injury = cruciate ligament sprain) but would be included in the numerator for the injury-specific annual incidence for both cruciate ligament sprain and meniscal tear. This method of annual cumulative incidence of persons injured, therefore, includes both preliminary and final diagnoses, and repeat as well as first injuries and would be expected to produce slightly higher estimates than previous studies estimating incidence of first injury based on final diagnosis (for example [11, 32]). No information is available on whether the left or right knee is affected for each injury. Individuals incurring an injury to their left knee and to their right knee in the same calendar year would be counted only once.

Annual incidence estimates were stratified by age (five-year age bands) and sex, and calculated separately for each of the nine calendar years between 2004 and 2012. For the same period 2004 to 2012, we estimated the mean number of soft-tissue knee injury consultations per calendar month to explore possible seasonal variations. Analyses were conducted using SAS software version 9.2 and 9.3. (SAS Institute Inc, Cary, NC, USA).

## Results

### Annual incidence estimates for clinically diagnosed soft-tissue knee injury

No trends in annual incidence estimates were evident on visual inspection (data not shown) and so we present crude mean annual cumulative incidence for the period 2004 to 2012.

**Figure 1:**
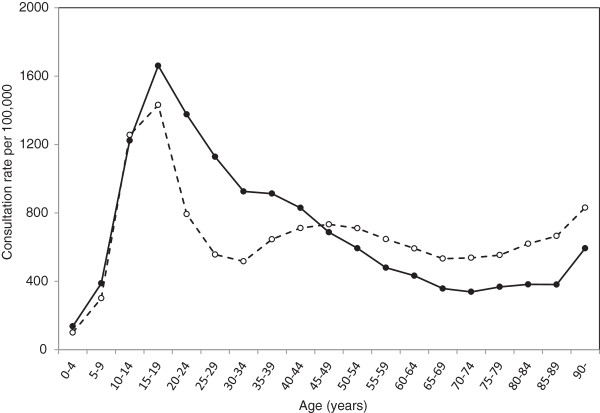
**Annual incidence for clinically diagnosed soft-tissue knee injuries among men (closed circles) and women (open circles): Skåne Healthcare Register, 2004 to 2012.**

The estimated annual incidence of persons sustaining any clinically diagnosed soft-tissue knee injury for the whole population was 720 per 100,000 persons (95% confidence interval (CI): 701, 739), and was higher in males than in females (Table 1).For males and females, the peak rate occurred in 15- to 19-year-olds (1,698 per 100,000 males and 1,464 per 100,00 females, respectively) (Figure 1). In males this peak was followed by a decline until reaching a plateau at the age of 65 years. In females, the peak rate in 15- to 19-year-olds was followed by a sharp decline until the age of 35 years but, unlike males, this was followed by an increase in rates in 35 to 49 year-olds. From 50 years onwards the incidence was higher in women than in men. In the most elderly (90 years and over) we observed another increase in incidence.

**Table 1:** **Annual incidence** ^**a**^ **(per 100,000 persons) for clinically diagnosed soft-tissue knee injuries: Skåne Healthcare Register, 2004 to 2012**

Soft-tissue knee injury	ICD-10	Total	Men	Women
		Annual incidence (95% CI)	Annual incidence (95% CI)	Annual incidence (95% CI)
Contusion	S80.0	204 (199, 209)	192 (184, 201)	216 (209, 223)
Dislocation of patella	S83.0	41 (39, 44)	40 (37, 42)	43 (40, 45)
Dislocation of knee	S83.1	3 (2, 4)	4 (3, 5)	2 (2, 3)
Meniscal tear	S83.2	79 (63, 94)	98 (78, 118)	60 (48, 71)
Articular cartilage tear	S83.3	18 (17, 19)	22 (20, 24)	13 (12, 14)
Collateral ligament sprain/strain	S83.4	70 (66, 74)	81 (76, 86)	60 (55, 65)
Cruciate ligament sprain/strain	S83.5	71 (61, 80)	86 (74, 98)	56 (49, 63)
Other/unspecified sprain/strain	S83.6	230 (204, 257)	244 (218, 269)	218 (190, 246)
Injury to multiple structures	S83.7	149 (137, 160)	169 (152, 186)	128 (121, 136)
Any		720 (701, 739)	766 (742, 789)	676 (649, 702)

^a^Annual incidence of persons injured, that is, proportion of the registered population with at least one medical encounter in the calendar year at which soft tissue injury was diagnosed and recorded, expressed per 100,000 individuals. 95%CI, 95 percent confidence interval; ICD-10, International Classification of Diseases, 10th revision.

### Seasonal variation

**Figure 2:**
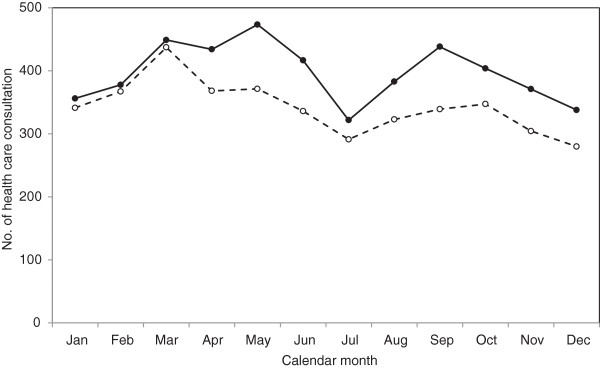
**Seasonal variation in clinically diagnosed soft-tissue knee injuries for men (closed circles) and women (open circles): Skåne Healthcare Register, 2004 to 2012.**

The incidence for clinically diagnosed soft-tissue knee injury was highest for males in the period between March to May, fell to its lowest rate in July before peaking again in September. In females, the peak rate was in March (Figure 2).

### Comparative age-sex distribution of annual incidence for different injury codes

**Figure 3:**
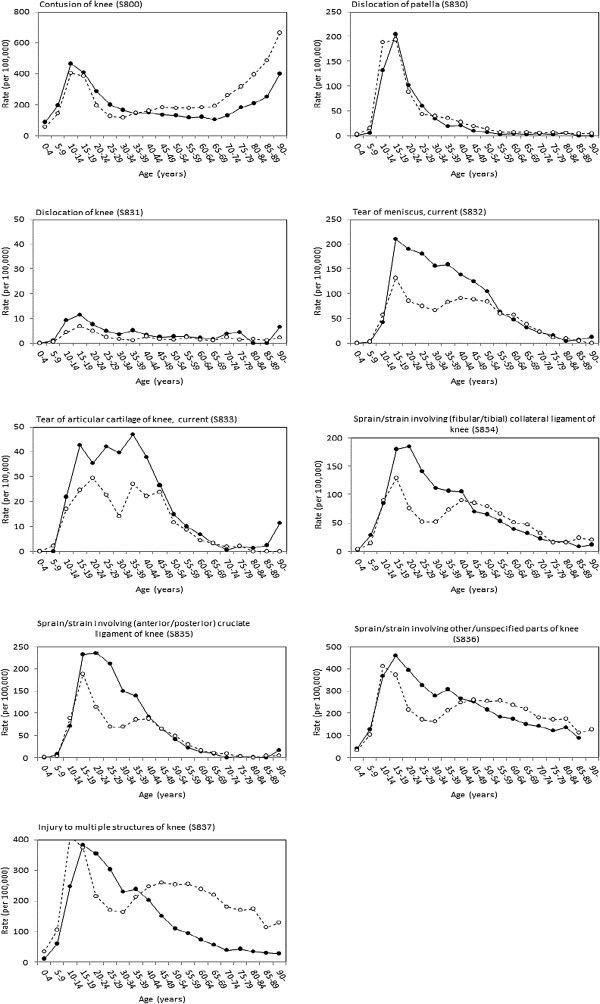
**Annual incidence for clinically diagnosed soft-tissue knee injuries among men (closed circles) and women (open circles), by injury code: Skåne Healthcare Register, 2004 to 2012.**

The distribution by age differed between injury codes (Figure 3; Additional file 1 provides the age-sex stratified incidence estimates plotted in Figure 3). There was a marked kurtosis in the age distribution for patellar dislocation around a sharp peak in 10- to 24-year-olds with rates below 10 per 100,000 from middle age onwards. In contrast, meniscal injury and sprains and strains declined only very gradually from peak rates in 15- to 19-year-olds. There was an exponential rise from the age of 65 years in the rate of contusion injuries but no corresponding rise in other soft-tissue knee injuries. In ligament, meniscal and multiple-structure injuries rates among females were lowest in the 20- to 34-year-old age range before rising again between the ages of 35 and 49 years. Males were at higher risk of ligament injury than females up to the age of 40 to 45 years.

## Discussion

This regional, population-wide study based on routinely recorded healthcare data from primary and secondary care over a nine-year period provides a broad description of the pattern of soft-tissue knee injuries presented to healthcare across the life course. Injury registries (for example, the Scandinavian ACL reconstruction registries [33]) and surveillance networks (for example [21]) are highly valuable sources of rich epidemiological data often on well-defined, specified injuries, in particular settings, or in special or high-risk populations. However, broader studies, such as the present one, are also justified on the grounds that knee injury represents a wide spectrum of exposure at multiple levels of the ‘injury pyramid’ (for example, hospitalisations, accident and emergency attendances, general practitioner visits, and those not presented to formal healthcare) [34, 35]. Minor injuries may be more prone than severe injuries to misclassification [35] and they may be less strongly related to future risk of osteoarthritis [2]. Nevertheless, we argue that they are still potentially relevant to understanding population exposure to injury and subsequent osteoarthritis development. Previous similarly broad population-wide studies have been either limited in size [11, 14, 36] or have focused on a particular class of injury [37].

Although based on routine recording as opposed to standardised, reference standard diagnostic criteria, our mean annual incidence estimates for clinically diagnosed soft tissue knee injury appear consistent with incidence rates from previous studies. Additional file 2 provides a data extraction table with key features in study design and published estimates from the current study and previous comparable studies. Our overall annual incidence (720 per 100,000) is higher than the incidence rate of 278.7 per 100,000 reported in the Olmsted County study. The Olmsted County study used a narrower case definition of new, isolated, acute injuries based on final diagnosis [11]. Conversely, our estimates are somewhat lower than the 1,080 and 1,160 per 100,000 estimates from studies conducted in Finland and Denmark, respectively, in the mid-1980s [14, 36]. Those previous studies included fractures as well as soft-tissue injuries.

Most comparable data exists for cruciate ligament injury. The observed peak age appears marginally younger in our study than in a national, population-wide study of ligament injuries from insurance data in New Zealand [37]. It is also younger than that reported in Scandinavian cruciate ligament reconstruction registries [38] and from cruciate ligament injury estimates from hospital in- and outpatient data in the Swedish National Patient Register [25]. It is possible that this may reflect earlier diagnoses recorded in primary care captured in SHR. Yet our annual incidence estimate for cruciate ligament sprain (71 per 100,000) closely approximated the incidence rate from Swedish hospital data [25]. They were also similar, within the 10- to 64-year age band, to the figure of 81 per 100,000 reported by Frobell *et al*. [24]. That study was based on injuries presenting to an emergency unit in the same geographical region but using magnetic resonance imaging confirmation of all suspected cases of acute anterior cruciate ligament injury. This peak of severe soft-tissue knee injuries in adolescence (10 to 19 years) and emerging adulthood (18 to 29 years) [39, 40] most likely is associated with sports participation. The substantial seasonal variation in injury rates, especially among men, would be consistent with this. Although the current study gathered no data on the inciting event or mechanism of injury, the peak months coincide with the skiing (March) and football seasons (spring-autumn) with dips in vacation periods and breaks in the sporting seasons.

Our study confirms and extends the distinctive age-related pattern in injury rates observed in women [9, 37] which show higher rates in adulthood after a decline from peak rates in late adolescence. In the present study, the increase was seen to begin in the age band 35 to 39 years and was evident across several injury codes, notably cruciate and collateral ligament sprains and injuries to multiple structures. In the same age band we observed a temporary interruption in the otherwise smooth decline in incidence rates among men. We speculate that this pattern of findings reflects an increased risk of injury during childrearing years (the mean maternal age at childbirth in Sweden is 31 years). A strong association between parity and subsequent risk of knee osteoarthritis has been reported in Danish registry data [41] and recently confirmed in US females in the Multicenter Osteoarthritis Study (MOST) cohort [42]. Injury was not taken into account in the former study and was not implicated as an important mechanism in the latter although this did rely on recall of lifetime injury at age 50 to 79 years. Injury risk in this stage of life may warrant closer investigation as part of a strategy for injury prevention in the general population.

A number of limitations in the present study should be noted. Firstly, we have not undertaken any independent verification of the accuracy of injury diagnoses within SHR although we have shown that our estimates for cruciate ligament injury are quite consistent with studies using more stringent reference standards [24]. However, this accuracy cannot be assumed to apply across all types of injury. Accurate diagnosis of soft-tissue knee injuries is challenging even in specialist settings. Frobell *et al*. [24] found, for example, that half of all patellar dislocations identified on MRI were missed by clinical examination in patients presenting to an orthopaedic emergency unit with acute torsional injury and rapid effusion. We would expect misclassification of diagnoses to affect estimates for specific injury codes more than the overall estimates for all soft-tissue knee injury codes combined. Nevertheless, healthcare records are likely to under-estimate the true occurrence of minor knee injuries [43]. This tendency toward conservative estimates could be exacerbated by restricting case ascertainment to those consulting and diagnosed by a physician although we found very few persons in a calendar year who had only consulted a non-physician for their knee injury (data not shown). Secondly, the lack of data on side-specific injury in the register data contributes to being unable to count more than one injury per person within the same calendar year. We, therefore, did not attempt to calculate an event rate based on injuries per person-time. The extent to which person-based incidence rates will be lower than these event rates depends on what proportion of injured individuals experience a recurrent or contralateral injury within the same calendar year. For severe soft-tissue knee injuries this rate of contralateral injury is likely to be low: previous studies suggest between 0.4% and 2.5% of individuals with an anterior cruciate ligament injury would experience the same injury on the contralateral knee [44]. To our knowledge there are no reliable estimates of recurrence and contralateral injury for all of the other types of knee injury we have investigated. Thirdly, our estimates are averages of rates for the period 2004 to 2012. However, we found no strong evidence of a trend in injury rates between 2004 and 2012 on visual inspection. During this period, the resident population in Skåne region increased by 9% (compared with 6% for Sweden as a whole) driven mainly by net immigration from abroad [45]. No dramatic changes in age composition were seen and, therefore, we think it is unlikely that important underlying changes in injury rates were masked by our approach to use average rates. Further, during the time period there was, to the best of our knowledge, no shift in policy, diagnostic coding or methods of diagnostics that would substantially have influenced case ascertainment. Fourthly, we did not include fractures in our estimates. These have been the subject of previous reports but are clearly also relevant to future osteoarthritis risk. Within the Skåne Healthcare Register, the annual incidence rates of fractures of the distal femur, patella and proximal tibia/fibula are, respectively, 13, 25 and 36 per 100,000 persons 20 years old and older (Rosengren B *et al*., submitted manuscript). Finally, our data are from Southern Sweden and their generalizability to other populations cannot be assumed. Regional data on lifestyle factors associated with the incidence of knee injury is limited but Sweden has higher than average rates of educational attainment [46] and relatively high levels of regular physical activity and sports participation compared with other European countries [47]. Although obesity has been increasing over recent decades [48, 49], the prevalence of obesity in Sweden has historically been in the lowest tertile internationally [50]. Further, the adjustment for missing data to private care is a crude adjustment as the potential influence of socioeconomic status and profession for knee injury and health care seeking behaviour is not accounted for.

Our findings carry several implications for understanding osteoarthritis risk. Firstly, severe knee injury such as cruciate ligament injury, while known to be a potent risk factor for osteoarthritis, is nevertheless a relatively infrequent exposure (71 per 100,000 persons per year) compared with the incidence rate of diagnosed knee osteoarthritis (650 per 100,000 persons per year) [51]. Causal mechanisms made up of other components must be responsible for a large proportion of osteoarthritis cases, even allowing for ligament injuries not captured in routine healthcare. The effect of minor injuries on osteoarthritis risk remains a challenge for epidemiologic research in this field. They are more numerous in the population than severe injury but are subject to greater misclassification (for example, due to inaccurate recall or being recorded as ‘flare-ups’ of osteoarthritis in older adults). Injuries are potentially recurrent events. One advantage of reporting annual incidence estimates as opposed to first-ever incidence rates is that one can see clearly the continued presentation of acute meniscal tears, and unspecified and superficial injuries into middle and old age. One question that arises from this is whether minor injury after osteoarthritis disease initiation and diagnosis contributes to the progression of osteoarthritis. Finally, the empirical induction period [52] between severe injury and osteoarthritis diagnosis may be several decades. The peak age for severe knee injury is 15 to 19 years while the incidence of diagnosed knee osteoarthritis continues to increase to age 75 years [51]. Such long intervals are beyond the current scope of electronic healthcare databases and injury registries. This fact is an argument in favour both of their long-term investment and in alternative study designs using markers of disease initiation as earlier outcome measurement.

## Conclusions

Clinically diagnosed soft tissue knee injury occurs with marked age and seasonal variations. The incidence of soft-tissue knee injury peaks in adolescence and emerging adulthood. As the potential induction point for many cases of post-traumatic knee osteoarthritis, injury prevention and early rehabilitation in this age group is justifiable. However, a range of knee injuries continue to occur across the adult lifespan including at ages when osteoarthritis is typically diagnosed and managed. The potential cumulative effect on osteoarthritis progression of these injuries may warrant further investigation.

## Electronic supplementary material

Additional file 1: **Age-sex specific mean annual incidence (per 100,000) for clinically diagnosed soft-tissue knee injuries, Skåne Healthcare Register, 2004 to 2012.** This table in pdf format provides the age-sex specific point estimates that are plotted in Figure 3. (DOCX 26 KB)

Additional file 2: **Comparison of current study estimates with other previously published population estimates of soft-tissue knee injuries.** This table in pdf format provides a data extraction table for the current study and previously published studies to enable comparisons to be made. (DOCX 19 KB)

## Abbreviations

ACL: anterior cruciate ligament
ICD: International Classification of Diseases
SHR: Skåne Health Care Register.

## References

[1] Blagojevic M, Jinks C, Jeffery A, Jordan KP (2010). Risk factors for onset of osteoarthritis of the knee in older adults: a systematic review and meta-analysis. Osteoarthritis Cartilage.

[2] Muthuri SG, McWilliams DF, Doherty M, Zhang W (2011). History of knee injuries and knee osteoarthritis: a meta-analysis of observational studies. Osteoarthritis Cartilage.

[3] Bendele AM (2001). Animal models of osteoarthritis. J Musculoskelet Neuronal Interact.

[4] European Bone and Joint Health Strategies Project (2004). European Action Towards Better Musculoskeletal Health. A Public Health Strategy to Reduce the Burden of Musculoskeletal Conditions. Turning Evidence into Practice.

[5] Arthritis Research Foundation and Centers for Disease Control and Prevention (2010). A National Public Health Strategy for Osteoarthritis.

[6] Englund M, Roos EM, Lohmander LS (2003). Impact of type of meniscal tear on radiographic and symptomatic knee osteoarthritis: a sixteen-year followup of meniscectomy with matched controls. Arthritis Rheum.

[7] Stein V, Li L, Lo G, Guermazi A, Zhang Y, Kent Kwoh C, Eaton CB, Hunter DJ (2012). Pattern of joint damage in persons with knee osteoarthritis and concomitant ACL tears. Rheumatol Int.

[8] Caine DJ, Golightly YM (2011). Osteoarthritis as an outcome of paediatric sport: an epidemiological perspective. Br J Sports Med.

[9] Day L, Valuri G, Ozanne-Smith J (1999). General practice injury surveillance in the LaTrobe Valley. Monash Univ Acad Res Center.

[10] Schappert SM, Burt CW (2006). Ambulatory care visits to physician offices, hospital outpatient departments, and emergency departments: United States, 2001–02. ital Health Stat 13.

[11] Yawn BP, Amadio P, Harmsen WS, Hill J, Ilstrup D, Gabriel S (2000). Isolated acute knee injuries in the general population. J Trauma.

[12] Kannus P, Niittymaki S, Jarvinen M, Lehto M (1989). Sports injuries in elderly athletes: a three-year prospective, controlled study. Age Ageing.

[13] Gage BE, McIlvain NM, Collins CL, Fields SK, Comstock RD (2012). Epidemiology of 6.6 million knee injuries presenting to United States emergency departments from 1999 through 2008. Acad Emerg Med.

[14] Nielsen AB, Yde J (1991). Epidemiology of acute knee injuries: a prospective hospital investigation. J Trauma.

[15] Kuikka PI, Pihlajamaki HK, Mattila VM (2013). Knee injuries related to sports in young adult males during military service - incidence and risk factors. Scand J Med Sci Sports.

[16] Clayton RA, Court-Brown CM (2008). The epidemiology of musculoskeletal tendinous and ligamentous injuries. Injury.

[17] Parkkari J, Pasanen K, Mattila VM, Kannus P, Rimpela A (2008). The risk for a cruciate ligament injury of the knee in adolescents and young adults: a population-based cohort study of 46 500 people with a 9 year follow-up. Br J Sports Med.

[18] Louw QA, Manilall J, Grimmer KA (2008). Epidemiology of knee injuries among adolescents: a systematic review. Br J Sports Med.

[19] Kujala UM, Taimela S, Antti-Poika I, Orava S, Tuominen R, Myllynen P (1995). Acute injuries in soccer, ice hockey, volleyball, basketball, judo, and karate: analysis of national registry data. BMJ.

[20] Hootman JM, Dick R, Agel J (2007). Epidemiology of collegiate injuries for 15 sports: summary and recommendations for injury prevention initiatives. J Athl Train.

[21] Swenson DM, Henke NM, Collins CL, Fields SK, Comstock RD (2012). Epidemiology of United States high school sports-related fractures, 2008–09 to 2010–11. Am J Sports Med.

[22] Mattila VM, Parkkari J, Korpela H, Pihlajamaki H (2006). Hospitalisation for injuries among Finnish conscripts in 1990–1999. Accid Anal Prev.

[23] Jones BH, Canham-Chervak M, Canada S, Mitchener TA, Moore S (2010). Medical surveillance of injuries in the u.s. Military descriptive epidemiology and recommendations for improvement. Am J Prev Med.

[24] Frobell RB, Lohmander LS, Roos HP (2007). Acute rotational trauma to the knee: poor agreement between clinical assessment and magnetic resonance imaging findings. Scand J Med Sci Sports.

[25] Nordenvall R, Bahmanyar S, Adami J, Stenros C, Wredmark T, Felländer-Tsai L (2012). A population-based nationwide study of cruciate ligament injury in Sweden, 2001–2009: incidence, treatment, and sex differences. Am J Sports Med.

[26] Ratzlaff CR, Liang MH (2010). New developments in osteoarthritis. Prevention of injury-related knee osteoarthritis: opportunities for the primary and secondary prevention of knee osteoarthritis. Arthritis Res Ther.

[27] Kuikka PI (2012). Epidemiology and magnetic imaging-based diagnostics of knee injuries and anterior knee pain in young adults.

[28] van Staa TP, Dennison EM, Leufkens HG, Cooper C (2001). Epidemiology of fractures in England and Wales. Bone.

[29] Englund M, Jöud A, Geborek P, Felson DT, Jacobsson LT, Petersson IF (2010). Prevalence and incidence of rheumatoid arthritis in southern Sweden 2008 and their relation to prescribed biologics. Rheumatology (Oxford).

[30] Haglund E, Bremander AB, Petersson IF, Strombeck B, Bergman S, Jacobsson LT, Turkiewicz A, Geborek P, Englund M (2011). Prevalence of spondyloarthritis and its subtypes in southern Sweden. Ann Rheum Dis.

[31] Jöud A, Petersson IF, Englund M (2012). Low back pain: epidemiology of consultations. Arthritis Care Res (Hoboken).

[32] Gabriel SE, Amadio PC, Ilstrup DM, Harmsen WS, Huschka TR, Hill JL, Yawn BP (2000). Change in diagnosis among orthopedists compared to non-orthopedists in the management of acute knee injuries. J Rheumatol.

[33] Granan LP, Bahr R, Steindal K, Furnes O, Engebretsen L (2008). Development of a national cruciate ligament surgery registry: the Norwegian National Knee Ligament Registry. Am J Sports Med.

[34] Polinder S, Haagsma JA, Toet H, van Beeck EF (2012). Epidemiological burden of minor, major and fatal trauma in a national injury pyramid. Br J Surg.

[35] Alexandrescu R, O'Brien SJ, Lecky FE (2009). A review of injury epidemiology in the UK and Europe: some methodological considerations in constructing rates. BMC Public Health.

[36] Kannus P, Jarvinen M (1989). Incidence of knee injuries and the need for further care. A one-year prospective follow-up study. J Sports Med Phys Fitness.

[37] Gianotti SM, Marshall SW, Hume PA, Bunt L (2009). Incidence of anterior cruciate ligament injury and other knee ligament injuries: a national population-based study. J Sci Med Sport.

[38] Granan LP, Forssblad M, Lind M, Engebretsen L (2009). The Scandinavian ACL registries 2004–2007: baseline epidemiology. Acta Orthop.

[39] Arnett JJ (2000). Emerging adulthood. A theory of development from the late teens through the twenties. Am Psychol.

[40] Arnett JJ, Booth A, Brown SL, Landale NS, Manning WD, McHale SM (2012). New horizons in research on emerging and young adulthood. Early Adulthood in a Family Context. National Symposium on Family Issues.

[41] Jørgensen KT, Pedersen BV, Nielsen NM, Hansen AV, Jacobsen S, Frisch M (2011). Socio-demographic factors, reproductive history and risk of osteoarthritis in a cohort of 4.6 million Danish women and men. Osteoarthritis Cartilage.

[42] Wise BL, Niu J, Zhang Y, Felson DT, Bradley LA, Segal N, Keysor J, Nevitt M, Lane NE (2013). The association of parity with osteoarthritis and knee replacement in the Multicenter Osteoarthritis Study. Osteoarthritis Cartilage.

[43] Walsh SS, Jarvis SN (1992). Measuring the frequency of “severe” accidental injury in childhood. J Epidemiol Community Health.

[44] Swärd P, Kostogiannis I, Roos H (2010). Risk factors for a contralateral anterior cruciate ligament injury. Knee Surg Sports Traumatol Arthrosc.

[45] Statistics Sweden (2014). Population statistics.

[46] OECD (2013). Education at a Glance 2013: OECD Indicators.

[47] TNS Opinion & Social (2010). Sport and Physical Activity.

[48] Neovius M, Teixeira-Pinto A, Rasmussen F (2008). Shift in the composition of obesity in young adult men in Sweden over a third of a century. Int J Obes (Lond).

[49] Neovius K, Johansson K, Kark M, Tynelius P, Rasmussen F (2013). Trends in self-reported BMI and prevalence of obesity 2002–10 in Stockholm County, Sweden. Eur J Public Health.

[50] Neovius M, Janson A, Rossner S (2006). Prevalence of obesity in Sweden. Obes Rev.

[51] Prieto-Alhambra D, Judge A, Javaid MK, Cooper C, Diez-Perez A, Arden NK (2014). Incidence and risk factors for clinically diagnosed knee, hip and hand osteoarthritis: influences of age, gender and osteoarthritis affecting other joints. Ann Rheum Dis.

[52] Rothman KJ (1981). Induction and latent periods. Am J Epidemiol.

