# Supplementary material for: Population-wide incidence estimates for soft tissue knee injuries presenting to healthcare in southern Sweden: data from the Skåne Healthcare Register
Source: Arthritis Res Ther. 2014 Jul 31;16(4):R162. doi: 10.1186/ar4678 (PMC4262192; doi:10.1186/ar4678)
Supplement: Supplementary file 1 — Additional file 1: Age-sex specific mean annual incidence (per 100,000) for clinically diagnosed soft-tissue knee injuries, Skåne Healthcare Register, 2004 to 2012. This table in pdf format provides the age-sex specific point estimates that are plotted in Figure 3. (DOCX 26 KB) [file 13075_2014_4351_MOESM1_ESM.docx]

**Additional File 1.** Age-sex specific mean annual incidence (per 100,000) for clinically diagnosed soft-tissue knee injuries, Skåne Healthcare Register, 2004-2012

|  | Contusion of knee (S800) | | | Dislocation of patella (S83.0) | | | Dislocation of knee (S83.1) | | | Tear of meniscus, current injury (S83.2) | | | Tear of articular cartilage of knee, current injury (S83.3) | | | Sprain/strain involving (fibular/tibial) collateral ligaments (S83.4) | | | Sprain/strain involving (anterior/posterior) cruciate ligaments (S83.5) | | | Sprain/strain involving other/unspecified parts of knee (S83.6) | | | Injury to multiple structures of knee (S83.7) | | |
| --- | --- | --- | --- | --- | --- | --- | --- | --- | --- | --- | --- | --- | --- | --- | --- | --- | --- | --- | --- | --- | --- | --- | --- | --- | --- | --- | --- |
| Age | M | F | Tot | M | F | Tot | M | F | Tot | M | F | Tot | M | F | Tot | M | F | Tot | M | F | Tot | M | F | Tot | M | F | Tot |
| 0-4 | 86 | 56 | 71 | 1 | 3 | 2 | 0 | 0 | 0 | 1 | 1 | <1 | - | - | - | 2 | 3 | 2 | 0 | 1 | 0 | 40 | 33 | 37 | 12 | 7 | 9 |
| 5-9 | 196 | 142 | 170 | 6 | 15 | 10 | 1 | <1 | <1 | 3 | 3 | 3 | 0 | 2 | 1 | 28 | 14 | 21 | 7 | 3 | 5 | 127 | 105 | 116 | 59 | 44 | 52 |
| 10-14 | 464 | 401 | 434 | 131 | 188 | 159 | 9 | 4 | 7 | 42 | 57 | 49 | 22 | 17 | 20 | 85 | 90 | 88 | 71 | 88 | 79 | 368 | 411 | 389 | 246 | 240 | 243 |
| 15-19 | 407 | 387 | 397 | 205 | 194 | 200 | 11 | 7 | 9 | 210 | 132 | 172 | 43 | 25 | 34 | 179 | 129 | 155 | 232 | 188 | 210 | 459 | 372 | 417 | 381 | 343 | 362 |
| 20-24 | 285 | 196 | 240 | 101 | 88 | 94 | 8 | 5 | 6 | 190 | 85 | 137 | 35 | 29 | 32 | 185 | 75 | 130 | 235 | 113 | 174 | 394 | 215 | 304 | 355 | 180 | 267 |
| 25-29 | 200 | 127 | 164 | 60 | 44 | 52 | 5 | 3 | 4 | 180 | 75 | 128 | 42 | 23 | 32 | 141 | 52 | 97 | 211 | 70 | 142 | 324 | 170 | 248 | 303 | 128 | 217 |
| 30-34 | 165 | 115 | 141 | 33 | 39 | 36 | 3 | 2 | 3 | 155 | 67 | 112 | 40 | 14 | 27 | 111 | 52 | 82 | 149 | 70 | 110 | 277 | 164 | 222 | 230 | 115 | 174 |
| 35-39 | 141 | 146 | 144 | 19 | 35 | 27 | 5 | 1 | 3 | 158 | 83 | 121 | 47 | 27 | 37 | 106 | 74 | 90 | 140 | 86 | 113 | 306 | 212 | 260 | 237 | 155 | 197 |
| 40-44 | 147 | 161 | 154 | 21 | 27 | 24 | 3 | 3 | 3 | 138 | 90 | 115 | 38 | 22 | 30 | 105 | 90 | 98 | 91 | 87 | 89 | 266 | 247 | 257 | 201 | 158 | 180 |
| 45-49 | 136 | 180 | 158 | 9 | 19 | 14 | 3 | 2 | 2 | 125 | 88 | 107 | 26 | 24 | 25 | 70 | 85 | 77 | 65 | 64 | 65 | 251 | 260 | 255 | 150 | 159 | 154 |
| 50-54 | 131 | 178 | 154 | 7 | 13 | 10 | 3 | 1 | 2 | 105 | 83 | 94 | 15 | 12 | 13 | 66 | 80 | 73 | 42 | 49 | 45 | 216 | 254 | 235 | 110 | 148 | 129 |
| 55-59 | 115 | 177 | 146 | 3 | 7 | 5 | 3 | 3 | 3 | 63 | 59 | 61 | 10 | 9 | 9 | 54 | 66 | 59 | 22 | 29 | 26 | 183 | 256 | 219 | 94 | 122 | 108 |
| 60-64 | 120 | 183 | 152 | 4 | 7 | 5 | 2 | 1 | 2 | 48 | 58 | 53 | 7 | 4 | 5 | 39 | 51 | 45 | 14 | 16 | 15 | 175 | 237 | 206 | 73 | 92 | 82 |
| 65-69 | 103 | 190 | 148 | 2 | 7 | 5 | 2 | 1 | 1 | 32 | 38 | 35 | 3 | 3 | 3 | 32 | 47 | 39 | 9 | 11 | 10 | 150 | 219 | 185 | 56 | 68 | 62 |
| 70-74 | 128 | 258 | 198 | 4 | 5 | 4 | 4 | 2 | 3 | 22 | 23 | 23 | 1 | 2 | 1 | 22 | 32 | 28 | 1 | 9 | 5 | 143 | 181 | 163 | 39 | 55 | 48 |
| 75-79 | 183 | 316 | 257 | 3 | 6 | 5 | 4 | 1 | 3 | 15 | 11 | 13 | 2 | 2 | 2 | 17 | 15 | 16 | 4 | 4 | 4 | 120 | 171 | 148 | 43 | 54 | 49 |
| 80-84 | 208 | 396 | 321 | 5 | 5 | 5 | 0 | 2 | 1 | 4 | 9 | 7 | 1 | 0 | 0 | 17 | 16 | 16 | 1 | 0 | 0 | 136 | 174 | 159 | 34 | 38 | 36 |
| 85-89 | 252 | 487 | 406 | 0 | 5 | 3 | 0 | 1 | 1 | 6 | 4 | 5 | 2 | 0 | 1 | 9 | 24 | 19 | 0 | 5 | 3 | 90 | 114 | 105 | 30 | 42 | 38 |
| 90- | 398 | 664 | 594 | 0 | 4 | 3 | 7 | 2 | 3 | 12 | 0 | 3 | 11 | 0 | 3 | 12 | 20 | 18 | 16 | 4 | 7 | 126 | 128 | 127 | 28 | 26 | 27 |
