# Supplementary material for: Population-wide incidence estimates for soft tissue knee injuries presenting to healthcare in southern Sweden: data from the Skåne Healthcare Register
Source: Arthritis Res Ther. 2014 Jul 31;16(4):R162. doi: 10.1186/ar4678 (PMC4262192; doi:10.1186/ar4678)
Supplement: Supplementary file 2 — Additional file 2: Comparison of current study estimates with other previously published population estimates of soft-tissue knee injuries. This table in pdf format provides a data extraction table for the current study and previously published studies to enable comparisons to be made. (DOCX 19 KB) [file 13075_2014_4351_MOESM2_ESM.docx]

**Additional File 2.** Comparison of current study estimates with other previously published population estimates of soft-tissue knee injuries

| Lead author, publication year | Location, date, size of catchment pop, sampling | Age range | Setting for case ascertainment | Injury types | Method of ascertainment | Estimates |
| --- | --- | --- | --- | --- | --- | --- |
| Kannus, 1989[36] | Orivesi Region, Finland  1985-1986 N=13,700  Census | All ages | Community health centre (outpatient and inpatient units) | All (soft-tissue and #) | Bespoke form completed by experienced general practitioners. Diagnosis per routine (Xray used on 21% injury visits, knee puncture 17%, arthroscopy 4%; 11.1% led to surgical consultation) | Overall: **1080 per 100,000 py**  Ligament injuries = 464/100,000 Contusions = 259/100,000  Meniscus = 216/100,000  Patella subluxation = 32/100,000 Knee # = 22/100,000  Seasonal variation: Peak visits in Mar (40), Jul (40), Aug (40), Jan (36), Dec (32) |
| Yawn, 2000[29] | Olmsted County, USA 1993-1996 N≈238 000  Census | 18+ | All medical facilities in Olmsted County (Mayo Clinic + Olmsted Medical Center) | Isolated, acute, new soft tissue injury (+ osteochondral #)  Exclusions: injuries limited to skin, evidence of chronic/system condition affecting knee (OA, RA), severe multisystem trauma | As per routine practice but also review of medical records to apply a 1-year look forward for final diagnosis (grouped into: (i) specific intra-articular = meniscus, cruciate lig, osteochondral #;  (ii) specific extra-articular = collateral lig, patella;  (iii) minor or other unspec = contusion, sprains) | Overall: **278.7 per 100,000 py** (F=243.2/100,000; M=311.0/100,000)  Meniscal/cruciate ≈ 48/100,000(?)  Collateral/patellar ≈69/100,000(?)  Sprain/strain ≈ 99/100,000(?) |
| Nielsen & Yde, 1991[11] | Aarhus, Denmark  1986 N=253,753  Census | All | Two emergency departments | Unclear  (included contusions, distortions, cruciate, collateral, meniscus, PF dislocation, PF overuse, #, overuse, others) | Diagnoses as per routine practice by any of: clinical examination, examination under anaesthesia, arthroscopic examination, surgery. 18 month look forward period used for final diagnosis | Overall: **1160 per 100,000 py** (F=970/100,000; M=1330/100,000)  Estimates from graph: M0-9=600;M10-19=2800;M20-29=2000;M30-39=1300;M40-49=1400;M50-=500; F0-9=400; F10-19=2300;F20-29=1300;F30-39=800;F40-49=800;F50-=600)  Male Female Total  Contusions 610 440 520  Distortions 300 190 240  Collateral 100 50 70  Cruciate 40 20 30  Meniscus 100 60 70  PF dislocation 20 50 30  PF overuse 30 50 40  # 30 40 40  Peak visits in Jan |

| Frobell, 2007[23] | Helsingborg, Sweden, 2002 N≈110,399  Census | 10-64 | Single emergency unit | Acute ACL | MRI verified | **81 per 100,000 py** |
| --- | --- | --- | --- | --- | --- | --- |
| Clayton, 2008[14] | Edinburgh, Scotland  1996-2001  N≈535,000  Census | 13-89 | Single orthopaedic trauma unit | Specific ligament or tendon lesions (including meniscus, cruciate, collateral, quads tendon rupture, patellar tendon rupture) | As per routine practice | Overall: ≈ **39.6 per 100,000 py** (F=18.3/100000, M=63.2/100000)  Male Female Total  Meniscus (37.7) (11.3) 23.8  ACL (12.9) (3.7) 8.1  Med coll (8.3) (2.5) 5.2  Lat coll (0.4) (0.0) 0.2  PCL repair (0.8) (0.1) 0.5  Quads tend rup (2.6) (0.5) 1.4  Pat tend rup (1.1) (0.3) 0.7 |
| Parkkari, 2009[15] | National, Finland  1987-2001  N=46,531  (Adolescent Health & Lifestyle Survey) | 14- 18 | National Hospital Discharge register | Cruciate ligament injury (ACL and PCL combined) | As per routine practice. Diagnostic codes: ICD-9 8442A, 8442B and ICD-10 S83.5 | Overall: Incidence density ≈ **60.9 per 100,000 py** (F=30.0/100000, M=96.6/100000) |
| Gianotti, 2009[38] | National, New Zealand, 2000-2005 N≈4,100,000  Census | All | Various (>30,000 registered medical professionals) | Knee ligament injuries (cruciate, collateral, meniscal, other ligament) | ACC claims database.  Injuries classed as non-surgical (NS), anterior cruciate ligament surgeries (ACLS), and other knee ligament surgeries (OKLS) | Overall: ≈ **1193.1 per 100,000 py**  Non-surgical = 1147.1 per 100,000 Anterior cruciate ligament surgery = 36.9 per 100,000  Other knee ligament surgeries = 9.1 per 100,000 |
| Current study | Skåne county, Sweden 2004-2012 N≈1,300,000  Census | All | Public sector primary and secondary care medical facilities | Soft-tissue knee injuries (excluding #) | As per routine practice. Diagnostic codes: ICD-10 S80.0, S83.0, S83.1, S83.2, S83.3, S83.4, S83.5, S83.6, S83.6P, S83.7 | Overall: **720 per 100,000 py** (F=657/100,000, M=759/100,000)  Contusion = 204 per 100,000  Dislocated patella = 41 per 100,000  Dislocated knee = 3 per 100,000  Meniscal tear = 79 per 100,000  Articular cartilage tear = 18 per 100,000  Collateral ligament sprain/strain = 70 per 100,000  Cruciate ligament sprain/strain = 71 per 100,000  Other sprain/strain = 230 per 100,000  Multiple = 149 per 100,000 |

ACC Accident Compensation Corporation; py person years; F female; M male; men/cru meniscus or cruciate ligament injury; coll/pat collateral ligament or patellar injury; spr/st sprain or strain
